# The Role of Cobalt Ions in Angiogenesis—A Review

**DOI:** 10.3390/ijms26157236

**Published:** 2025-07-26

**Authors:** Wiktor Gregorowicz, Lukasz Pajchel

**Affiliations:** 1 Students Scientific Group NANODRUG, Faculty of Pharmacy, Medical University of Warsaw, Banacha 1 Str., 02-091 Warsaw, Poland; s083169@student.wum.edu.pl; 2 Department of Pharmaceutical Chemistry and Biomaterials, Faculty of Pharmacy, Medical University of Warsaw, Banacha 1 Str., 02-091 Warsaw, Poland

**Keywords:** cobalt, angiogenesis, biomaterials

## Abstract

Cobalt is an essential trace element involved in key biological processes. It serves most notably as a component of vitamin B_12_ (cobalamin) and a regulator of erythropoiesis. While cobalt deficiency can lead to disorders such as megaloblastic anemia, excess cobalt poses toxicological risks to the thyroid, cardiovascular, and hematopoietic systems. In recent years, cobalt ions (Co^2+^) have gained attention for their ability to mimic hypoxia and promote angiogenesis. This represents a crucial mechanism for tissue regeneration. Cobalt mediates this effect mainly by stabilizing hypoxia-inducible factor 1α (HIF-1α) under normoxic conditions, thereby upregulating angiogenic genes, including VEGF, FGF, and EPO. Experimental studies—from cell culture to animal models—have demonstrated cobalt-induced enhancement of endothelial proliferation, migration, and microvascular formation. Emerging evidence also indicates that Co^2+^-stimulated macrophages secrete integrin-β1-rich exosomes. These exosomes enhance endothelial motility and tubulogenesis independently of VEGF. Furthermore, cobalt-modified biomaterials have been developed to deliver cobalt ions in a controlled manner. Examples include cobalt-doped β-tricalcium phosphate or bioactive glasses. These materials support both angiogenesis and osteogenesis.This review summarizes current findings on cobalt’s role in angiogenesis. The emphasis is on its potential in cobalt-based biomaterials for tissue engineering and regenerative medicine.

## 1. Introduction

Angiogenesis is defined as the formation of new blood vessels from pre-existing vasculature. It is a highly regulated and complex process that plays a crucial role in human physiology. Angiogenesis plays a pivotal role in various physiological and pathological conditions. Angiogenesis begins early in embryonic development and continues throughout life. It contributes to processes such as tissue growth, wound healing, and reproductive function. However, while the physiological roles of angiogenesis are widely acknowledged, its involvement in pathological conditions must not be underestimated. A prominent example of pathological angiogenesis is the neovascularization observed in tumor development. This process facilitates tumor growth, survival, and metastasis. It thereby significantly contributes to disease progression and worsens patient prognosis [1,2,3].

A comprehensive understanding of the molecular mechanisms governing both the stimulation and suppression of angiogenesis is essential. This knowledge advances targeted therapeutic approaches in regenerative medicine and cancer treatment. Among the numerous factors that modulate angiogenic activity, certain metallic elements, particularly cobalt, copper, and titanium, have attracted increasing attention. Copper and cobalt are essential trace elements that participate in a wide range of biochemical and physiological functions, while titanium is extensively used in biomedical implants and regenerative materials due to its biocompatibility.

Cobalt serves as a central component of vitamin B12 (cobalamin). This vitamin is crucial for DNA synthesis, red blood cell production, and the maintenance of neurological function. A deficiency in cobalt—manifesting as vitamin B12 deficiency—can lead to megaloblastic anemia and neurological impairments. These include cognitive dysfunction and motor coordination disorders [1,2,3].

The angiogenic potential of cobalt ions is especially noteworthy. Although not commonly recognized in clinical practice as a pro-angiogenic agent, the role of cobalt in promoting neovascularization is well-documented in scientific literature. A search for the phrase “cobalt in angiogenesis” in the PubChem database returns 353 articles. This highlights a growing interest in the role of cobalt in vascular biology. These entries span peer-reviewed research articles, chemical substance data, and pathway-related information. Notably, the number of publications on this topic has been steadily increasing year by year. This reflects a rising recognition of cobalt’s influence on angiogenic mechanisms.

This has encouraged us to explore and organize the existing knowledge on the angiogenic mechanisms of cobalt. We focus particularly on its potential biomedical applications. Despite the substantial number of publications addressing cobalt’s pro-angiogenic properties, no comprehensive review article has yet been published on this topic.

In this study, we specifically focus on the pro-angiogenic properties of cobalt ions. We also critically address their potential cytotoxicity, which represents a significant barrier to clinical use. A growing body of evidence has confirmed that cobalt ions can stimulate angiogenesis through various molecular pathways. Furthermore, recent studies suggest that incorporating cobalt ions into biomaterials used in tissue engineering and regenerative medicine can help harness their angiogenic potential while reducing cytotoxic effects. This strategy allows for localized delivery, controlled release, and reduced systemic exposure, thereby enhancing biocompatibility and therapeutic efficacy [1,4,5,6,7,8,9,10,11,12,13,14,15].

## 2. Angiogenesis

### 2.1. Initiation of Angiogenesis

**Figure 1:**
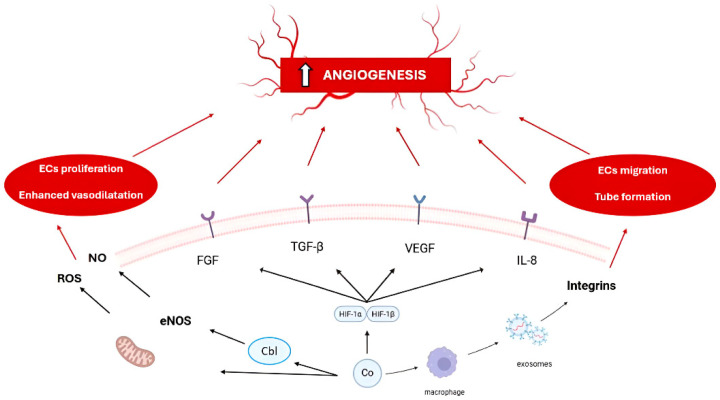
Selected mechanisms of angiogenesis initiation by cobalt.

Angiogenesis is the physiological process of forming new blood vessels from pre-existing vasculature. The process occurs through endothelial cell proliferation, migration, and tube formation. It is regulated by pro-angiogenic factors (e.g., vascular endothelial growth factor (VEGF), fibroblast growth factor (FGF), and hypoxia-inducible factor (HIF-1α)) and their inhibitors. Oxygen and vital nutrients are transported through the vascular system. The system also facilitates immune monitoring. The inner vessel walls are lined with endothelial cells (ECs), which actively promote tissue regeneration and development. Stressors such as mechanical damage, tumor formation, or hypoxia can induce and modulate the intensity of this process. Initially, acidic and basic fibroblast growth factors (aFGF/bFGF) were thought to be the primary drivers of angiogenesis. Over time, studies revealed a broader spectrum of angiogenic regulators. These include VEGF, TGF-β, angiogenin, angiopoietins, and mast-cell-derived tryptase [16]. VEGF belongs to the PDGF growth factor family and demonstrates strong angiogenic capacity. Under controlled laboratory conditions, VEGF exhibits direct mitogenic effects on endothelial cells from arterial, venous, and lymphatic vascular origins [17]. Tissue hypoxia represents the key regulatory signal for this growth factor. It is abundantly expressed by epidermal cells in wound healing contexts [18]. HIF-1 is present in two oxygen-sensitive HIF-1α and constitutive HIF-1β subunits. It serves as the master regulator of hypoxic responses. Under hypoxic conditions, impaired hydroxylation of HIF-1α prevents its degradation. This allows nuclear translocation, where it dimerizes with HIF-1β to activate transcription of hypoxia-responsive genes, including VEGF [19]. This mechanism leads to a cascade of processes forming new blood vessels. It is mostly controlled by oxygen levels, but it can also be manipulated by metal ions, which mimic the state of hypoxia. This thereby induces angiogenesis [3,20]. In Figure 1, we present an overview of the established signaling pathways, growth factors, and receptors involved in modulating angiogenesis induced by cobalt.An additional regulatory element involved in the modulation of angiogenesis is endothelial nitric oxide synthase (eNOS) and inducible nitric oxide synthase (iNOS) which plays a critical role in the process by modulating endothelial cell behavior through nitric oxide (NO) signaling. NO is a key mediator of vascular homeostasis and neovascularization, influencing processes such as endothelial cell proliferation, migration, tube formation, and survival.

The study demonstrates that eNOS regulates sprouting angiogenesis and retinal neovascularization by controlling the polarization of endothelial cells. This is achieved through the modulation of the actin cytoskeleton and intercellular junctions, particularly via the regulation of vascular endothelial cadherin (VE-cadherin) and Rho GTPases (RhoA and Rac1). Proper activation and spatial distribution of these molecules are essential for directed cell migration and the formation of new vascular sprouts [21].

In vitrostudies have shown that short-term exposure of endothelial cells to 50–100 μM cobalt chloride (CoCl_2_) stabilizes HIF-1α, promoting the expression of pro-angiogenic genes. While the direct activation of the PI3K/Akt → PKA →eNOS cascade by cobalt remains to be fully elucidated, there is evidence that Co^2+^ ions can enhance NO production through indirect mechanisms. Leonard et al. (1998) demonstrated that cobalt ions generate reactive oxygen species (ROS), including superoxide (O_2_^−^), hydrogen peroxide (H_2_O_2_), and hydroxyl radicals (•OH), via Fenton-like reactions, particularly in the presence of biological chelators, such as glutathione and anserine [22]. These ROS may act as secondary messengers that modulate redox-sensitive signaling pathways, potentially influencing eNOS activity. Additionally, other studies suggest that cobalamin (Co^3+^) may help maintain tetrahydrobiopterin (BH_4_) availability and prevent eNOS uncoupling, thereby supporting efficient NO synthesis [21]. Collectively, these mechanisms indicate that cobalt can enhance NO bioavailability, facilitating endothelial cell migration and tube formation and linking the Co^2+^–HIF axis with classical NO-dependent angiogenic pathways [23].

### 2.2. Trace Elements Supporting Angiogenesis

**Figure 2:**
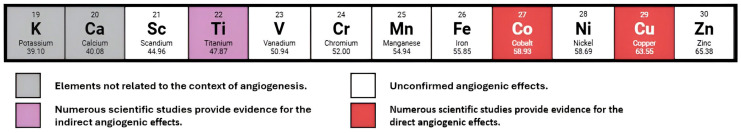
The imageis a segment of Group 4 of the periodic table, with the metals that have been identified as possessing pro-angiogenic properties highlighted [3].

Many studies have shown the potential role of trace elements in angiogenesis mechanisms. They improve these mechanisms by interacting with growth factors, enzymes, and signaling pathways. For example, the review conducted by the research team from the University of Zürich provides a comprehensive summary of evidence. This evidence shows that titanium (Ti), copper (Cu), and cobalt (Co) ions have strong influence on angiogenesis, as illustrated in Figure 2. Although many transition metals have their role in this mechanism, the three mentioned are found to have the most impact. Thisisconfirmed by agreat number of studies [3].

#### 2.2.1. Titanium

Titanium is not indispensable in the body. However, due to its exceptional corrosion resistance and biocompatibility, titanium has found extensive use in medicine. It is used to manufacture orthopedic and dental implants, as well as surgical components [2,3].

Research has shown that Ti surface topography can modulate pro-angiogenic factor production in osteoblasts through integrin-dependent mechanisms. Integrins, which are present on ECs, play a key role in activating intracellular signaling cascades. These pathways control the upregulation of growth factor receptors, including VEGF receptors. These ultimately promote blood vessel formation. The titanium alloy substrate stimulated markedly higher production of pro-angiogenic factors. These include VEGF-A, fibroblast growth factor (FGF)-2, and angiogenic growth factor (ANG)-1. Experimental evidence demonstrates that Ti-conditioned media significantly enhance EC activation. Thisupregulates key angiogenic markers—notably VEGFR-1, VEGFR-2, and both eNOS and iNOS.

Of particular importance, eNOS-derived NO production critically supports endothelial monolayer formation and expansion. Furthermore, studies have shown that titanium’s role in enhancing ECs’ proliferation is more noticeable when its surface is roughened [24]. This results in confirmed superior cell proliferation, adhesion, and increased eNOS/VEGFR production versus control conditions. However, the relationship between Ti surface topography and upregulation of angiogenic factors requires further investigation [25].

#### 2.2.2. Copper

Copper is an essential trace element serving as a critical cofactor in numerous enzymatic reactions fundamental to human physiology. It plays a key role in iron transport and absorption, thereby supporting hemoglobin synthesis and overall erythropoiesis. In addition, copper contributes to the development and maintenance of connective and bone tissues. It participates in melanin synthesis, influencing skin and hair pigmentation. Copper also supports immune system function and exerts antioxidative effects. It neutralizes free radicals and protects cells from oxidative stress. Deficiency in copper can lead to anemia, growth retardation, compromised immune responses, and neurological dysfunctions [1,3].

At the molecular level, copper-dependent enzymes facilitate a wide range of biological processes. These include mitochondrial oxidative phosphorylation, cellular defense against ROS, regulation of coagulation pathways, and promotion of angiogenesis. Copper enhances EC migration and proliferation, partly by upregulating VEGF expression. This regulation is influenced by ROS, such as hydrogen peroxide (H_2_O_2_), which stimulate VEGF production. This process is further amplified by copper’s catalytic participation in the Haber–Weiss reaction. Experimental studies demonstrate significantly enhanced VEGF expression in keratinocytes co-treated with H_2_O_2_ and copper sulfate. This was compared to cells exposed to H_2_O_2_ alone. Furthermore, copper modulates angiogenesis by interacting with multiple regulatory factors, including HIF-1α, angiogenin, FGF-1, and eNOS.

In addition to its pro-angiogenic and regenerative properties, copper exhibits notable antibacterial and antiseptic activity. This is particularly relevant in wound healing applications. These combined biological effects suggest that copper incorporation into biomedical implants may offer considerable therapeutic benefits, enhancing tissue regeneration while simultaneously minimizing infection risk [26].

#### 2.2.3. Cobalt

Cobalt, to which we would like to devote the most attention in this study, has extensively described effects on the angiogenesis process. This is documented in a great number of publications. Although not all reviewed studies met predefined inclusion criteria, several provided compelling evidence supporting the role of cobalt in promoting angiogenesis.

Co is known for HIF-1α stabilization by mimicking the hypoxia state in tissue, which leads to VEGF upregulation. Angiogenesis is strongly influenced by hypoxic conditions. During microvascular remodeling, hypoxia activates macrophages. These, in turn, secrete pro-angiogenic factors, such as VEGF-A [27].

Cobalt exhibits a dualistic biological role, acting both as a pro-oxidant metal capable of generating ROS, and as an essential cofactor in the form of cobalamin (vitamin B12), which supports nitric oxide synthase (NOS) activity, particularly the endothelial isoform eNOS.

On one hand, free cobalt ions (Co^2+^) are known to catalyze the formation of ROS through redox cycling. Cobalt can mediate the one-electron reduction of molecular oxygen, producing superoxide anions (O_2_^−^), which subsequently dismutate into hydrogen peroxide (H_2_O_2_). In the presence of reducing agents or chelators, cobalt participates in Fenton-like reactions, generating hydroxyl radicals (•OH), leading to oxidative damage of cellular components, including DNA, proteins, and lipids [22]. ROS produced in tissues can positively influence angiogenesis. At moderate levels, ROS act as signaling molecules that support the formation of new blood vessels, particularly during tissue repair and remodeling. This stimulatory effect highlights their important role in physiological vascular responses [23,28].

When cobalt is incorporated into cobalamin, it plays a protective and regulatory role, supporting a range of cellular functions, including NO synthesis [29]. More on this topic is discussed in Section 3.2, Roles of Cbl.

Cobalt also exerts a significant influence via epigenetic regulation. Recent findings indicate that cobalt–chromium (Co–Cr) alloys modulate the angiogenic response of endothelial cells by altering DNA methylation and histone modification patterns. Specifically, Co–Cr exposure engages DNA methyltransferases—primarily DNMT3B—and demethylating enzymes, such as TET1 and TET2, thereby shifting the balance of DNA methylation and impacting the transcriptional landscape of angiogenesis-related genes. Additionally, histone deacetylase HDAC6, which regulates microtubule polymerization and cytoskeletal dynamics, appears to be strongly involved in shaping the migratory and proliferative behavior of endothelial cells under Co–Cr influence. The activation of SIRT1 further supports cobalt’s epigenetic role by modulating HIF-1α expression in hypoxic-like microenvironments, enhancing endothelial survival and promoting angiogenic signaling. Collectively, these mechanisms provide a novel understanding of how cobalt may epigenetically steer endothelial phenotype and vessel formation, offering important insights into the success of osseointegration and the vascular integration of implantable biomaterials [30].

Among these elements, cobalt ions deserve special attention. An increasing number of studies report their pro-angiogenic effects and the growing potential for therapeutic applications. Their use is primarily considered in implantology. As a component of biomaterials, cobalt exhibits excellent properties in enhancing both angiogenesis and osteogenesis. This improves treatment efficacy and reduces treatment time. In this article, the impact of Co in the mechanism of angiogenesis and its role in the proper functioning of human physiology will be considered in greater detail. Furthermore, the potential application of this trace element in implantology and bone tissue regeneration—particularly through its role in promoting angiogenesis—will also be examined.

The table below (Table 1) summarizes the angiogenic mechanisms of the selected metals. It includes their associated molecular targets and toxicity profiles. Cobalt and copper exhibit direct pro-angiogenic activity through well-characterized molecular pathways involving HIF-1α and VEGF. Titanium acts more indirectly, primarily through surface interactions that support endothelial cell function. Cobalt is particularly notable for its ability to simulate hypoxia, a key driver of angiogenesis. Copper plays a supportive biochemical role as a cofactor. Titanium’s role is more structural and context-dependent, relying on biomaterial design. Toxicity profiles also differ significantly, with cobalt and copper requiring dose control. Titanium is considered highly biocompatible.

## 3. The Biological Role of Cobalt in the Human Body and Its Toxicity

### 3.1. Organic Form of Cobalt in Physiology and Its Sources

**Figure 3:**
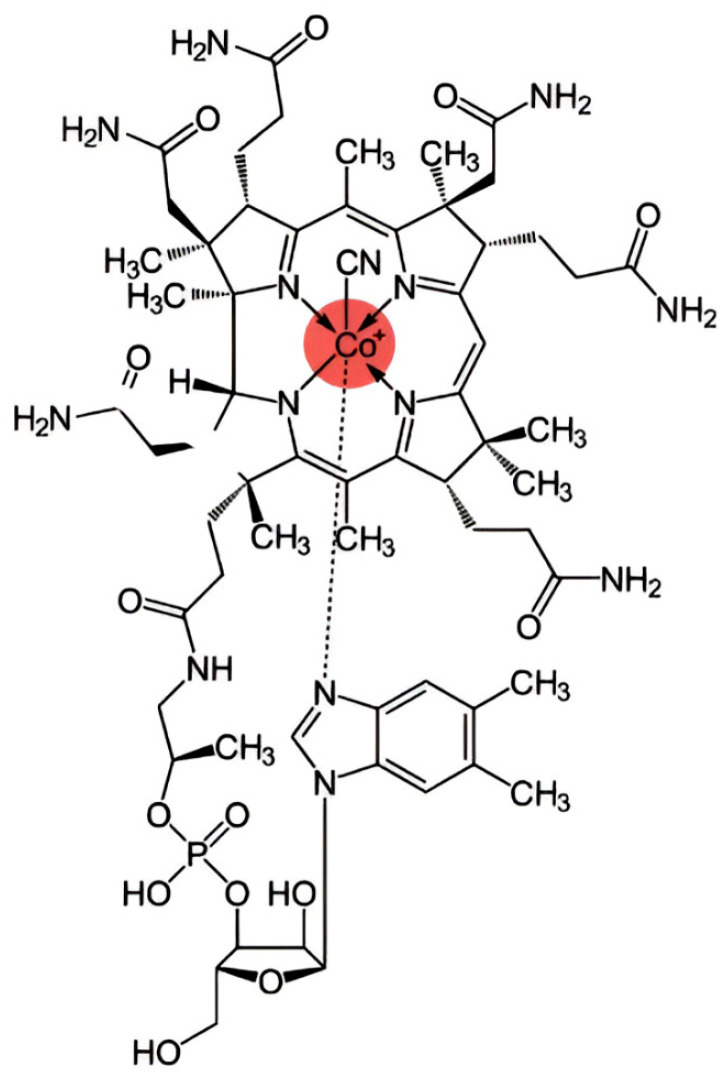
The structural formula of cobalamin is presented, with the cobalt ion highlighted.

Cobalt is a trace element that plays a fundamental role in human physiology. This occurs through its incorporation into (Figure 3) cobalamin (Cbl), which is known as vitamin B_12_. Although ionic cobalt itself does not perform direct biological functions in human cells, it is indispensable. This is due to its central position within the corrin ring of Cbl. In this position, it can exist in multiple valence states, most notably Co^3+^ and Co^2+^. These are involved in key enzymatic reactions.

Cobalt ions, although not considered essential in free ionic form for human physiology, are taken up by cells through transporters that primarily mediate the uptake of other divalent metal ions. In human cells, Divalent Metal Transporter 1 (DMT1, SLC11A2) is a key transporter expressed in the duodenum, erythroid cells, and other tissues, and is known to facilitate the uptake of Co^2+^ alongside Fe^2+^ under conditions of metal deficiency or stress [31]. It is possible that ZIP8 (SLC39A8), a known transporter of divalent metal ions such as Zn^2+^, Mn^2+^, and Cd^2+^, may also facilitate the uptake of Co^2+^ under certain experimental conditions. While direct evidence in human physiology remains limited, in vitro studies—particularly those using heterologous expression systems—have indicated that ZIP8 can mediate cobalt transport [32].

Additionally, cobalt is delivered indirectly as part of the cobalamin transport system, involving intrinsic factor, cubilin, and transcobalamin II (TCN2), with receptor-mediated endocytosis in tissues with high proliferative activity. The expression of these transporters is regulated by metal availability, inflammatory signals, and cellular oxygen status [33]. Collectively, these systems enable both the regulated uptake and intracellular routing of cobalt, either as ionic Co^2+^ or incorporated within cobalamin.

The dietary daily intake of cobalt varies between 5 and 50 μg per day [34]. The recommended allowance of Cbl for the adult human is 2.4 μg per day [35]. Cbl is synthesized by microorganisms in the stomach or can be delivered through dietary intake of animal-derived foods. The concentration of vitamin B_12_ in most foods is minimal. While it is typically lacking in fruits and vegetables, nori, a type of edible seaweed, is a notable plant-derived source containing a substantial amount of vitamin B_12_. Nevertheless, the bioavailability of B_12_ from seaweed remains a subject of debate. Certain seaweed species may contain pseudo-vitamin B_12_, an inactive analog of the vitamin. Since humans require the biologically active form of Cbl, the human body has evolved a highly selective and specialized mechanism to accurately recognize and absorb only the authentic form of the vitamin [36].

### 3.2. Roles of Cbl

Cbl functions as a coenzyme in two essential metabolic reactions: the remethylation of homocysteine to methionine and the isomerization of L-methylmalonyl-CoA to succinyl-CoA. These enzymatic processes are crucial for hematopoiesis and the maintenance of neurological integrity [37]. Cobalamin plays a crucial role in DNA biosynthesis and serves as a cofactor for two key enzymes: methylmalonyl-CoA mutase, an enzyme involved in the catabolic pathway of odd-chain fatty acids via β-oxidation, and methionine synthase. Vitamin B_12_ plays a critical role in myelin biosynthesis, which is essential for the formation of the myelin sheath—a lipid-rich layer enveloping neuronal axons that functions as an electrical insulator. This enhances the speed and efficiency of nerve impulse conduction. Its involvement in both myelin formation and remyelination is a key aspect for neuronal repair and regeneration following nerve injury [38].

The most well-defined clinical outcome of vitamin B_12_ deficiency is megaloblastic anemia, also referred to as pernicious anemia. This is believed to result from methionine synthase inactivation [39]. Related macrocytosis associated with vitamin B_12_ or folate deficiency arises from ineffective or abnormal erythropoiesis. This reflects impaired nuclear maturation during red blood cell development. Both nutrients serve as essential cofactors in DNA synthesis and cellular proliferation, and their deficiency disrupts the normal maturation process across multiple cell lineages [40]. Other characteristic symptoms of its deficiency in organisms are methylmalonic aciduria and cobalamin neuropathy. The first mentioned is caused by a defect of methylmalonyl-CoA mutase (MCM). Multiple factors can contribute to its impaired function, including insufficient vitamin B_12_ intake, defects in cobalamin absorption or intracellular processing pathways, as well as mutations in the gene encoding the MCM enzyme [41,42]. Cobalamin-related neuropathy, on the other hand, is a peripheral nervous system disorder observed in individuals with vitamin B_12_ deficiency. Similar neurological impairments have been reported in patients exposed to nitrous oxide (N_2_O), implicating methionine synthase (MS) dysfunction in the pathogenesis of this condition. While disruption of methylation pathways is recognized as a contributing factor [43,44], the precise molecular mechanisms underlying Cbl neuropathy remain incompletely explained.

Studies show that cobalamin also plays an important role in angiogenesis. Vitamin B_12_ deficiency has been strongly correlated with elevated plasma homocysteine (Hcy) concentrations [45]. This is due to its critical role as a cofactor for methionine synthase, the enzyme responsible for the remethylation of Hcy to methionine [46]. When vitamin B_12_ is insufficient, the activity of methionine synthase is impaired, resulting in accumulation of Hcy in the bloodstream [47]. Studies on homocysteine cytotoxicity have demonstrated that elevated Hcy levels, particularly in the presence of adenosine (Ade), significantly reduce cell proliferation and viability and induce cell cycle arrest at the G1/S transition in endothelial cells [48]. Furthermore, the combination of Hcy and Ade was shown to impair endothelial cell migration and inhibit capillary-like tube formation. These are essential steps in angiogenesis. At the molecular level, this combination of Hcy and Ade leads to a downregulation of mRNA expression of key angiogenic regulators, including VEGF, VEGFR1, and VEGFR2. In addition, it results in decreased protein levels of VEGF, as well as components of the downstream signaling pathway, such as extracellular signal-regulated kinases 1 and 2 (ERK1/2). Collectively, these effects confer strong anti-angiogenic properties to elevated homocysteine caused by deficiency of Cbl. Additionally, several studies have identified an association between elevated plasma homocysteine levels (hyperhomocysteinemia) and a range of pathological conditions, including schizophrenia [49], Alzheimer’s disease [50], and osteoporosis [51].

Cobalamin also plays a key role in supporting nitric oxide synthesis through regulation of eNOS. Glutathionylcobalamin (GluGSCbl) has been shown to selectively enhance eNOS activity during early inflammatory responses by maintaining the availability of essential cofactors, such as tetrahydrobiopterin (BH_4_), nicotinamide adenine dinucleotide phosphate (NADPH), flavin adenine dinucleotide (FAD), flavin mononucleotide (FMN), and heme. This ensures proper, coupled eNOS function, which produces nitric oxide rather than superoxide. Moreover, cobalamin contributes to redox balance by sustaining intracellular glutathione (GSH) levels, thereby preventing eNOS uncoupling and limiting ROS formation. Additionally, GSCbl may function as a redox modulator and peroxynitrite scavenger, offering further protection to endothelial cells from nitrosative stress [29]. The results suggest that among other functions, vitamin B_12_ exhibits pro-angiogenic properties through multiple biochemical mechanisms, including the reduction of plasma Hcy concentrations. By facilitating NO production and mitigating homocysteine-induced endothelial dysfunction, vitamin B_12_ contributes to the promotion of angiogenesis and the maintenance of vascular health [52]. It is hypothesized that the pro-angiogenic properties attributed to cobalamin may, at least in part, be mediated by its cobalt ion, whose pro-angiogenic propertiesare well known and scientifically proven. The possible mechanisms by which cobalt could contribute to angiogenesis will be explored in more detail in a subsequent section of this article.

### 3.3. Toxicity of Cobalt

The primary routes of Co exposure in humans are through the inhalation of atmospheric air and the ingestion of food and drinking water contaminated with cobalt-containing compounds. Regulatory guidelines have established that cobalt concentrations in drinking water should not exceed 1–2 mg/L. The EPA has established a chronic reference dose (RfD) of 3 × 10^−4^ mg/kg/day based on decreased iodine uptake, with a no-observed-adverse-effect level (NOAEL) of 1 mg/kg/day [53]. The average daily cobalt intake in humans has been estimated to be 5–40 μg, with the geometric mean blood level for the U.S. population being 0.173 μg/L [54]

Consequently, the quantitative analysis and surveillance of cobalt levels in consumables is critical for safeguarding public health. This includes soft drinks and other food products [55]. The gastrointestinal absorption of inorganic cobalt compounds varies considerably, ranging from 5% to 45%, depending on their degree of solubility in water. Inhaled cobalt absorption ranges from 52% to 78%, while skin absorption varies, with <1% through intact skin and almost 80% through abraded skin [54]. In industrial or occupational settings, individuals are primarily exposed to cobalt through inhalation, particularly in the form of inorganic cobalt particles [45]. The American Conference of Governmental Industrial Hygienists (ACGIH) has established a threshold limit value (TLV) of 0.02 mg/m^3^ for occupational exposure to cobalt [53]. According to the French Agency for Food, Environmental, and Occupational Health and Safety (AFSSA), the acceptable daily intake for cobalt has been estimated at 1.6 to 8 micrograms per kilogram of body weight per day [55]. In addition, the International Council for Harmonisation (ICH), through its Q3D (R1) guidance, has specified a maximum permissible daily oral intake of cobalt at 50 micrograms per day.

Cobalt has been shown to exert toxicological effects on multiple organ systems. These include the thyroid gland, cardiovascular system, and hematopoietic tissues. Blood cobalt concentrations below 300 μg/L are not associated with any adverse effects in most individuals, while concentrations between 300 and 700 μg/L are associated with hematological effects and reversible endocrine reactions, including polycythemia and decreased iodine uptake. Blood cobalt concentrations of 700–800 μg/L and higher may pose risks of more serious neurological, reproductive, or cardiac effects [56]. Additionally, cobalt exposure has been implicated in occupational pulmonary disorders (cobalt asthma) [46], hypersensitivity reactions, and neoplastic processes [57]. The International Agency for Research on Cancer (IARC) has addressed the carcinogenic potential of cobalt. The IARC has classified cobalt metal and soluble cobalt(II) salts as Group 2A (probably carcinogenic to humans), while cobalt(II) oxide is classified as Group 2B (possibly carcinogenic to humans) [58,59]. The National Toxicology Program (NTP) has classified cobalt and cobalt compounds that release cobalt ions in vivo as reasonably anticipated to be human carcinogens [60].

While the toxicological profile summarized above largely follows Lison, it is important to note that more recent reviews address other relevant mechanisms and health risks [34,61]. For example, Keegan et al. (2008) provide a systematic comparison showing that wear debris and cobalt ion release from metal-on-metal orthopedic implants can serve as a significant source of systemic cobalt exposure [62]. These clinical sources can lead to local inflammation, implant loosening, and in some cases neurological or cardiomyopathic effects, even at moderate systemic blood cobalt concentrations, underscoring the importance of considering both environmental and implant-related risks. Likewise, Genchi et al. (2023) [63] detailed mechanisms of cobalt bioaccumulation in tissues (including bone, kidney, and liver) and highlighted the prolonged persistence of cobalt in the body due to its affinity for proteins, such as hemoglobin.

Cobalt particles can trigger immunological reactions and damage the local tissue. For example, inhalation of cobalt dust may impair lung function. Hard metal pneumoconiosis is a rare occupational lung disorder associated with exposure to cobalt-containing dusts, leading to progressive lung fibrosis and inflammation [64]. Nanoparticles released from metal-on-metal hip implants have been linked to inflammation and bone degradation [62]. Systemic cobalt toxicity can occur when Co^2+^ ions enter the bloodstream and lymphatic system, allowing distribution throughout the body. In vitro studies identify the ionized, “free” form of cobalt as the main contributor to its systemic toxic effects [65,66].

The precise pathways through which cobalt exerts its toxic influence remain incompletely understood. However, several proposed interactions may account for its cellular impact. Cobalt exhibits a strong chemical attraction toward sulfhydryl moieties. This potentially disrupts the functionality of key proteins by altering their thiol-dependent architecture, including those involved in mitochondrial bioenergetics [67]. It may also interfere with the structural integrity of metalloenzymes by substituting native divalent cations at their catalytic cores [35]. Additionally, cobalt has been implicated in modulating calcium signaling pathways. It acts as a blocker of calcium influx through membrane channels [68] and possibly restricts calcium mobilization and its downstream signaling events by competing with Ca^2+^ for binding to intracellular effector proteins [69]. Furthermore, cobalt is capable of triggering cellular oxidative stress via the formation of ROS. This likely occurs through Fenton-like chemical mechanisms, which can inflict molecular damage to DNA, structural proteins, and lipids [35]. Exposure to cobalt ions induces neurodegenerative damage by activating HIF-1α, which in turn leads to overproduction of ROS. The resulting oxidative stress impairs autophagic flux, disrupts mitochondrial homeostasis, and contributes to cellular energy deficits and neuronal cell death. These mechanisms, involving both ROS accumulation and autophagy deregulation, underline the role of cobalt in promoting neurotoxicity and degeneration of brain tissue [70].

A dangerous feature of cobalt is its ability to accumulate in the body. This leads to long-term negative health effects, especially as highlighted by Genchi et al. (2023) in their review of cobalt bioaccumulation in various tissues [63]. In human red blood cells, the membrane transport pathway responsible for cobalt uptake appears to be shared with calcium [71]. Studies have shown that Co slowly crosses the cell membrane into the cytoplasmic compartment. This process is effectively irreversible because cobalt is not actively extruded by the calcium pump and becomes tightly bound to the globin portion of hemoglobin. As a result, the concentration of free, ionized Co^2+^ in the cytoplasm represents only about 1% of the total cobalt concentration. Initially, cobalt binding to hemoglobin is reversible. However, over time, a significant and increasing fraction becomes tightly bound, likely due to oxidation of Co^2+^ to Co^3+^ [71]. The elimination of cobalt is represented as a multi-compartmental model, with compartments having half-lives of several hours to a week [54]. During chronic systemic exposure to cobalt in humans and laboratory animals, tissue accumulation occurs, particularly in the liver and kidneys [72]. While cobalt ions from blood accumulate in bone tissue to a limited degree, the scope of this problem increases when cobalt-containing biomaterials, such as bone- substitution implants, are present in the human body [73]. Due to Ca^2+^Co^2^ substitutional properties, some amounts of cobalt accumulate in bone tissue and can cause the abovementioned toxic effects. Micromolar concentrations of Co^2+^ delivered to developing osteoclast precursors increase both osteoclast differentiation and their resorptive function, with a maximum 75% increase in osteoclast numbers and 2.3- to 2.7-fold increase in mineral resorption observed with 0.1 μM Co^2+^ and 0.1–10 μM Co^2+^, respectively [74]. High concentrations, in addition, inhibit the proliferation of osteoblasts and HUVECs (human umbilical vein endothelial cells) [75]. Cobalt levels below 10 µg/mL appear to be tolerated by osteoblast cells, while higher concentrations reduce proliferation and osteoblast activities [76].

Additionally, cobalt compounds have been shown to induce significant alterations in histone post-translational modifications, thereby modulating the epigenetic landscape of exposed cells. Exposure to CoCl_2_ and cobalt oxide (Co_3_O_4_) results in increased trimethylation of histone H3 at lysine 4 (H3K4me3) and lysine 27 (H3K27me3), as well as changes in histone H4 acetylation. Mechanistically, cobalt inhibits the activity of iron-dependent histone demethylases, such as JMJD2A, by competitively binding to the catalytic site, leading to the accumulation of repressive methylation marks like H3K9me3 and H3K36me3. Moreover, the cobalt-induced elevation of H3K4me3 and H3K27me3 levels is dependent on methionine availability, suggesting that cobalt may also enhance the activity of histone methyltransferases or increase the supply of methyl donors. These findings indicate that cobalt disrupts chromatin homeostasis by simultaneously enhancing both activating and repressive histone marks, potentially leading to widespread transcriptional dysregulation. Such epigenetic imbalances may contribute to the toxic and carcinogenic effects associated with cobalt exposure [77].

It is worth noting that under some conditions (for example, ischemia and tissue hypoxia), cobalt’s effects may also have beneficial implications. Small amounts of cobalt and its controlled liberation can enhance many physiological processes mentioned above. This especially includes the improvement of angiogenesis. Co^2+^ upregulates VEGF secretion, with treatments at 50, 100, or 150 μM significantly increasing VEGF expression in vitro, although it also inhibits endothelial network formation in co-cultures [78].

However, the effect of cobalt on VEGF is not always linear or unequivocally positive: for example, Rana et al. (2019) demonstrated that CoCl_2_ increased the expression of HIF-1α and VEGF only up to a certain concentration, after which further increases led to reduced proliferation and increased expression of pro-apoptotic markers, such as p53 and BAX [79]. This reflects the presence of a biphasic, dose-dependent response, which should be considered when analyzing angiogenic or cytotoxic outcomes.

Lower Co^2+^ concentrations (5, 10, or 25 μM) maintain significantly upregulated VEGF expression while mitigating inhibitory effects on cell growth [78]. Cobalt stimulates VEGF mRNA expression in cardiac myocytes, with cobalt and manganese increasing VEGF mRNA 34.0 ± 22.0-fold and 10.8 ± 1.1-fold, respectively [80].

## 4. Angiogenic Properties of Cobalt

### 4.1. Role of Cobalt in Angiogenesis

We focused on publications that describe the effect of cobalt ions on the mechanism of angiogenesis and provide strong experimental evidence to support it. We analyzed 30 relevant studies.

An important role of cobalt ions in angiogenesis comes from their ability to activate hypoxia-inducible factor. This metal-induced HIF activation can trigger the expression of various hypoxia-responsive genes, potentially driving tumor formation and progression on a larger scale, but it has the potential to enhance angiogenesis under optimized levels. These genes are involved in these processes through angiogenic growth factors and erythropoiesis [81].

The diversity of cobalt-based compounds and biomaterials, along with their angiogenic mechanisms and biological effects, is summarized in Table 2. This table provides a concise overview of key studies and will serve as a reference point for the detailed discussion in the following sections.

As shown in Table 2, cobalt ions and cobalt-containing biomaterials exert their pro-angiogenic effects via multiple molecular pathways, with varying efficacy and cytotoxicity profiles depending on the compound and experimental model.

Through direct action on erythropoietin-producing cells in the kidneys and liver, cobalt is known to simulate hypoxic conditions. This occurs, triggering metal-induced activation of the HIF transcriptional pathway. This activation ultimately leads to the stimulation of erythropoietin gene expression. The erythropoietic response to cobalt is known to be intensified under hypoxic conditions [82]. Co^2+^ ions are believed to inhibit key oxygen-sensitive enzymes, including HIF-α prolyl hydroxylase (HIF-α-PH) and HIF-α asparaginyl hydroxylase [83]. Both act as crucial intracellular oxygen sensors during hypoxic signaling. As an inducer of the hypoxic response, cobalt triggers the coordinated activation of a wide array of HIF-dependent genes. Among these targets are genes involved in promoting angiogenesis. Examples include those encoding VEGF and angiopoietin, various glycolytic enzymes, regulators of iron metabolism and transport, as well as genes that govern apoptosis and cellular proliferation [35]. As mentioned above, hypoxic conditions activate macrophages, which in turn release a range of pro-angiogenic factors. Studies showed that exosomes secreted by macrophages influenced by cobalt in experimental conditions contained a number of modulators of angiogenesis, such as RNA, increasing expression of VEGF and eNOS. This mechanism was confirmed by in vitro and in vivo experiments with ECs treated with such exosomes. Treatment led to increased tube formation in endothelial cells. As mentioned earlier, ECs take part in the integrin-mediated mechanism of angiogenesis. Since integrin-β1 expression was at the highest level in the cobalt-exosome treated ECs, this experiment demonstrated the role of cobalt ions in supporting angiogenesis [27].

**Table 2:** Summary of key cobalt-based compounds and biomaterials investigated for their pro-angiogenic effects, including main molecular mechanisms, biological effects, and direct references from the review. The table covers both free cobalt ions and cobalt-releasing biomaterials, with emphasis on HIF-1α/VEGF pathway activation, macrophage/exosome involvement, and effects on osteogenesis and wound healing.

Cobalt Compound/Biomaterial	Experimental Model	Main Angiogenic Mechanism (s)	Biological Effect (s)	Sources
Cobalt chloride (CoCl_2_)	Endothelial cells, animal models	HIF-1α stabilization, ↑VEGF, ↑FGF, ↑SDF-1; mimics hypoxia	↑Migration, proliferation, tube formation, angiogenesis	[27,79,84,85,86,87,88,89]
Cobalt-doped bioactive glass	hBMSC, HUVEC, animal models	HIF-1α/VEGF, upregulation of bFGF, RUNX2, BMP-2	↑VEGF expression, angiogenesis, osteogenesis	[5,6,7,8,9,10,11,75]
Cobalt-doped mesoporous silica nanoparticles	rBMSC, rat	HIF-1α/VEGF, CD31 activation	↑VEGF secretion, neovascularization	[4,7]
Cobalt-containing borate glass fibers	HUVEC, wound model	HIF-1α/VEGF, upregulation of angiogenic proteins	↑Proliferation, migration, accelerated wound healing	[10,14]
Cobalt-doped calcium phosphate coatings	Goat (in vivo)	Upregulation of angiogenesis markers	↑Number and size of blood vessels in tissue	[15]
Cobalt protoporphyrin (CoPPIX)	HMEC-1	HO-1 (HIF-1-independent), ↑VEGF, ↑IL-8	↑VEGF and IL-8 expression	[88]
Cobalt nanowires (CoNWs)	HUVEC	HIF-1α stabilization	↑Endothelial cell proliferation	[12]
Free cobalt ions	Macrophages, endothelial cells	Integrin-β1-rich exosomes, ↑VEGF, ↑eNOS	↑Migration, ↑tube formation	[27]
Cobalt-substituted β-TCP ceramics	HUVEC, animal models	HIF-1α stabilization, VEGF upregulation	↑Angiogenesis, ↑VEGF expression	[13]
Cobalt-doped borosilicate bioactive glass	BMSC, animal models	HIF-1α/VEGF, SDF-1, BMP-2, RUNX2 upregulation	↑Angiogenesis, ↑osteogenesis	[90]

### 4.2. The Effect of Free Cobalt Ions and Cobalt Chloride on Angiogenesis

The research team of Zhang et al. demonstrated that cobalt ions affect macrophage behavior and endothelial cell function in several distinct ways. While Co^2+^ was shown to suppress macrophage proliferation in a concentration-dependent manner, it did not significantly alter exosome secretion by macrophages. It also did not affect the uptake of these vesicles by endothelial cells. Interestingly, the macrophage-derived exosomes enhanced endothelial cell migration and angiogenic activity both in vitro and in vivo. This pro-angiogenic effect is likely mediated by increased expression of NO, VEGF, and integrin-β1 in the ECs [27].

The researchers Rana et al. used PCR to assess gene expression levels and observed amplification products of the expected sizes for β-actin, HIF-1α, VEGF, p53, and BAX genes. Their results demonstrated that exposure to CoCl_2_ led to a dose-dependent increase in the expression of HIF-1α and VEGF in both MCF-7 and MDA-MB-231 cell lines. These findings provide strong experimental evidence that CoCl_2_ effectively simulates hypoxic conditions by upregulating hypoxia-responsive genes [79]. They observed that “CoCl_2_ induced HIF-1α accumulation and consequently upregulated the expression of VEGF up to a certain concentration, which confers the hypoxia induction at the respective concentrations”.

In a study investigating the effects of intravesical cobalt chloride infusion, Western blot analysis revealed that rat bladders treated with CoCl_2_ for six days exhibited significantly elevated levels of HIF-1 protein compared to saline-treated controls. When the same blot was re-probed for VEGF—a downstream target of HIF signaling—its expression was also markedly increased in cobalt-treated tissues. This indicates that CoCl_2_ stimulates the HIF-1/VEGF pathway in bladder tissue [84]. The positive effect of CoCl_2_ on the angiogenesis process was confirmed by the study in [85]. This study demonstrated that CoCl_2_ significantly enhances the migration, proliferation, and angiogenic potential of CD133^+^ cells under hypoxic conditions. Migration assays and wound healing experiments showed increased cell motility in response to CoCl_2_, while MTT assays indicated enhanced cell viability. ELISA and real-time PCR analyses revealed elevated expression of VEGF, FGF, SDF-1, and HIF-1α at both the protein and mRNA levels. These findings were further confirmed by Western blotting. This indicates that CoCl_2_ promotes angiogenesis-related pathways by stabilizing HIF-1α and upregulating downstream pro-angiogenic factors. Similar results were obtained in another study [86].

In another study using real-time qPCR analysis, bone-marrow-derived stem cells (BMSCs) exposed to CoCl_2_-induced hypoxic conditions exhibited significantly elevated VEGF gene expression compared to cells cultured under normoxic conditions. This upregulation was further confirmed at the protein level by Western blotting, which showed increased intracellular VEGF levels in the CoCl_2_-treated group. Additionally, subcutaneous implantation of materials revealed that constructs seeded with CoCl_2_-treated BMSCs attracted a greater number of blood vessels surrounding the implant compared to other groups. This observation was further supported by von Willebrand factor (vWF) staining, which confirmed a higher vascularization within the scaffolds containing CoCl_2_-treated cells [87]. Another study showed that Co-doped bioactive glass scaffolds—specifically those containing 0.5%, 1%, and 3% cobalt—significantly stimulated VEGF protein secretion in human bone-marrow-derived stem cells (hBMSCs). Additionally, HIF-1α expression in hBMSCs increased in correlation with the cobalt concentration in the scaffolds. The expression levels of angiogenic and osteogenic genes, including bFGF, VEGF, SDF-1, BMP-2, and RUNX2, were upregulated in hBMSCs at both incubation time points (7 and 14 days) [90].

In another study, the researchers extended their investigation beyond the commonly studied VEGF pathway by examining the involvement of additional angiogenic mediators. By including interleukin-8 (IL-8) alongside VEGF, they aimed to provide a more comprehensive understanding of the molecular mechanisms through which cobalt-based compounds influence angiogenesis. In this study, the authors investigated the mechanisms by which cobalt protoporphyrin (CoPPIX) and CoCl_2_ influence angiogenesis-related factors in human microvascular endothelial cells (HMEC-1). The results showed that CoPPIX induced heme oxygenase-1 (HO-1) expression and significantly upregulated VEGF and IL-8 via promoter activation. In contrast, CoCl_2_ enhanced VEGF expression through a HIF-1-dependent mechanism involving ROS, without affecting IL-8. The study highlights distinct signaling pathways for CoCl_2_ and CoPPIX, demonstrating that CoPPIX acts via HO-1 independently of HIF-1, while CoCl_2_ promotes angiogenesis through HIF-1 activation and ROS production. VEGF production in HMEC-1 cells was three-fold above basal levels. This upregulation was observed at both the protein and transcriptional levels, suggesting transcriptional control. Although both compounds enhanced VEGF transcription, only CoCl_2_ activated the hypoxia-responsive element (HRE) within the VEGF promoter, implicating HIF-1 involvement. In contrast, CoPPIX did not affect HRE activity, indicating an HIF-1-independent mechanism [88].

Another research team investigating the effect of cobalt on angiogenesis observed that cobalt administration in rats led to a marked increase in transgene expression throughout the kidney compared to controls. Quantitative PCR analysis confirmed a significant upregulation of HIF-1 and HIF-2 target genes. By the ninth week, mRNA levels of erythropoietin (EPO) and VEGF had increased markedly—more than three-fold and nearly two-fold, respectively—indicating a strong cobalt-induced activation of hypoxia-responsive gene expression [89].

The mechanisms of cobalt ion action in angiogenesis are summarized in Table 2 for better visualization.

The data summarized in Table 2 and Table 3 reveal critical dose-dependent effects of cobalt compounds. CoCl_2_ demonstrates optimal angiogenic efficacy at concentrations of 50–100 μM, as evidenced by significant VEGF upregulation and enhanced endothelial cell functions [78,79,85,86,87,90]. However, cytotoxicity concerns emerge at concentrations exceeding 200 μM, leading to reduced cell viability and pro-apoptotic responses. Importantly, similar therapeutic concentrations are observed regardless of the cobalt source, as demonstrated by comparable VEGF production between cells exposed to 100 μM CoCl_2_ and those treated with cobalt-containing biomaterials [5]. This consistency is further supported by studies showing that cobalt-doped bioactive glasses (2–5% doping), cobalt-substituted β-tricalcium phosphate, and cobalt-containing fibers all exhibit similar angiogenic efficacy within comparable effective concentration ranges [6,7,8,12,13,90]. The narrow therapeutic window (50–100 μM efficacy vs. >200 μM toxicity) underscores the importance of controlled delivery systems and precise dosing in cobalt-based therapeutic applications, while the similar effectiveness across different cobalt-containing materials suggests that the bioactive cobalt ions, rather than the carrier material, are the primary determinant of the angiogenic response. Cytotoxic effects at higher doses are further evidenced by cobalt-induced proliferation arrest in osteoblast-like cells, and increased osteoclast activation at sub-toxic micromolar concentrations [74,76].

### 4.3. The Effect of Cobalt-Containing Biomaterials on Angiogenesis

In the reviewed publications, the effects of cobalt ions, including both free ions and ions derived from cobalt chloride, on angiogenesis were investigated. As previously discussed, the direct action of cobalt, particularly at elevated concentrations, is highly toxic. Biomaterials serving as cobalt ion carriers appear to partially address this issue. Numerous studies have reported that cobalt retains its pro-angiogenic properties when gradually released at low concentrations from the surface of bioactive glasses, calcium phosphates, or other biomaterials, while it exhibits significantly reduced cytotoxicity.

Cobalt released from biomaterials has been extensively investigated for its potential applications in tissue engineering. An increasing number of studies provide evidence that such materials not only exhibit favorable physicochemical properties, they also possess pro-angiogenic and osteogenic capabilities, largely attributed to the biological activity of cobalt ions. A study by Zhao et al. demonstrated that cobalt-doped mesoporous silica-coated magnetic nanoparticles (Co-MMSNs) significantly promoted angiogenesis during distraction osteogenesis. In vitro, Co-MMSNs enhanced VEGF secretion by rBMSCs, with a notable increase observed after 72 h of exposure compared to both control and non-doped MSN groups. In vivo, immunohistochemical analysis revealed elevated expression of CD31—a marker of endothelial cells—in the regenerating bone tissue of rats treated with Co-MMSNs, indicating enhanced neovascularization within the distraction gap. These findings suggest that Co-MMSNs effectively stimulate angiogenic activity. This likely occurs through cobalt-mediated activation of hypoxia-inducible pathways. This contributes to improved vascularization and accelerates bone regeneration [4].

Research conducted by Anu K. Solanki et al. also demonstrated enhanced HIF-1α stabilization in fibroblasts following exposure to CoCl_2_ and bioactive glass containing cobalt. The degree of stabilization increased progressively from 2% to 5% cobalt concentration in bioactive glass and then CoCl_2_ treatment. As a downstream effect of HIF-1α activation, VEGF expression was significantly elevated in fibroblasts treated with either conditioned media or CoCl_2_ compared to the DMEM control. Interestingly, no notable difference in VEGF production was observed between cells exposed to 100 μM CoCl_2_ and those treated with the cobalt-containing media [5].

Studies show that adding Co^2+^ ions to cell cultures or releasing them from bioactive glass significantly boosts VEGF production at both the genetic and protein levels. Researchers also found that implanting cells exposed to cobalt onto collagen scaffolds improved the growth of new blood vessels within bone tissue. This effect likely occurs because cobalt tricks cells into behaving as if they are in a low-oxygen environment. It does this by stabilizing HIF-α, a key hypoxia-sensitive protein, which then activates genes involved in blood vessel development [6]. The authors of this study referred to findings from previous publications that support this mechanism, using existing scientific evidence to substantiate their own results. One such study showed that the incorporation of 2% cobalt into mesoporous bioactive glass (MBG) scaffolds led to a notable increase in VEGF secretion by BMSCs after seven days. Additionally, the expression of HIF-1α in BMSCs rose in correlation with the cobalt content. Both 2% and 5% cobalt-doped MBG scaffolds significantly upregulated VEGF gene expression compared to undoped MBG and control samples [7]. The effect of cobalt-containing bioactive glass (Co-BG) in promoting angiogenesis through both in vitro and in vivo mechanisms was also confirmed in another study [8]. In vitro assays showed that Co-BG did not impair HUVEC viability, while it significantly enhanced tube formation and upregulated VEGF-A and HIF-1 gene expression, indicating pro-angiogenic activity at the molecular level. Similar conclusions were drawn by researchers in other studies [1,9,10], where this property was shown to be enhanced when combined with other ions in cooperation with cobalt. Expanding on this, one study [11] also demonstrated associated osteogenic properties.

A study conducted by Zhao et al. showed that cobalt nanowires (CoNWs) enhanced the proliferation of HUVECs, as evidenced by increased absorbance values in CCK-8 assays. HUVECs cultured in the leachate derived from CoNWs exhibited greater growth compared to those in standard DMEM, particularly on day 5. This suggests that the cobalt ions released from CoNWs facilitate endothelial cell proliferation. The authors attribute this effect to the well-established role of cobalt ions in mimicking hypoxic conditions. Cobalt ions can substitute for iron in the heme group, thereby stabilizing HIF-1α [12]. A similar outcome was observed in another study [13], where enhanced angiogenesis and increased VEGF expression were also demonstrated for a biomaterial doped with Co ions, in this case TCP.

Research conducted by Zhang et al. revealed that the release of Co^2+^ ions from cobalt-containing fibers enhanced the proliferation, migration, and tube formation of HUVECs by triggering a hypoxic response and upregulating key angiogenic proteins, including HIF-1α and VEGF. This study noted that re-epithelialization—the process by which a wound is covered with new epithelial tissue—is crucial in the wound healing process, and the completion of re-epithelialization is often used as a key indicator of successful wound closure. In vivo animal tests in this study demonstrated that wounds treated with Co-containing fibers healed more completely compared to those in the control and borate bioactive glass groups [14].

An interesting study was conducted by Tahmasebi et al., in which the formation of new blood vessels was observed during the 12-week implantation period. This study showed that embedding Co^2+^ ions into a calcium phosphate (CaP) coating led to the development of more numerous and larger blood vessels following intramuscular implantation in goats. Histological and immunohistochemical evaluations revealed that polylactic acid (PLA) particles coated with CaP containing Co^2+^ ions exhibited a greater number and larger area of blood vessels compared to both uncoated PLA particles and those coated with CaP without cobalt. While small vessels were present across all samples, large blood vessels were primarily observed in the cobalt-containing group. These findings further support the use of Co^2+^-doped CaP coatings to promote in vivo vascularization [15].

## 5. Conclusions and Future Perspectives

Cobalt ions have emerged as potent regulators of angiogenesis, primarily through the stabilization of HIF-1α under normoxic conditions. This leads to the upregulation of key pro-angiogenic mediators, such as VEGF, FGF, and EPO. The optimal angiogenic window for cobalt compounds appears to lie between 50 and 100 μM, while concentrations exceeding 200 μM are associated with cytotoxic effects. Cobalt-containing biomaterials offer promising platforms for localized, controlled release, enabling therapeutic efficacy while minimizing systemic toxicity.

Despite significant progress, several critical knowledge gaps hinder clinical translation. These include incomplete understanding of cobalt’s interaction with eNOS, insufficient data on long-term dose–response relationships, lack of validated biomarkers for patient stratification and treatment monitoring, and limited insight into the relationship between cobalt release kinetics and local angiogenic outcomes. Furthermore, the long-term safety profile of cobalt-based therapies in humans remains inadequately characterized.

Additional limitations must also be addressed to ensure safe and effective clinical application. In vivo toxicological concerns, such as potential bioaccumulation and the influence of specific cobalt forms and biomaterial compositions, require thorough investigation. Moreover, the lack of standardized methodologies for dose quantification across experimental models hampers result comparability and complicates the translation of preclinical findings into clinical practice. While cobalt-based strategies show promise in promoting angiogenesis, long-term safety remains a critical consideration. The potential for cobalt accumulation in tissues, interference with metal homeostasis, and its classification by the IARC as a possible human carcinogen (Group 2B) underscore the need for comprehensive toxicological assessments.

Mechanistic studies elucidating cobalt-eNOS signaling and downstream pathways are essential to identify novel therapeutic targets. Ultimately, well-designed clinical trials are needed to evaluate safety and efficacy in specific indications, such as wound healing, tissue regeneration, and ischemic diseases.

Emerging technologies, such as smart biomaterials or 3D-printed scaffolds, could offer new opportunities for precise, localized cobalt delivery. Incorporating cobalt into these platforms can minimize systemic exposure while maximizing therapeutic benefit. These interdisciplinary innovations position cobalt as a promising component in next-generation regenerative strategies.

In conclusion, cobalt-based approaches hold significant potential for therapeutic angiogenesis. Their ability to mimic hypoxic signaling and activate multiple pro-angiogenic pathways positions them as valuable tools in regenerative medicine. Realizing this potential will require sustained collaboration among materials scientists, molecular biologists, and clinicians to address safety, efficacy, and translational challenges.

## Figures and Tables

**Table 1:** Molecular pathways and angiogenic effects of selected metallic elements.

Element	Primary Mechanism of Action in Angiogenesis	Key Molecular Targets/Pathways	Notes on Toxicity
Co	Mimics hypoxia and stabilizes HIF-1α, leading to VEGF upregulation and macrophage-mediated secretion of pro-angiogenic factors	↑ HIF-1α→↑ VEGF-A; macrophage activation; angiogenic cytokine secretion	Dose-dependent cytotoxicity; embedding in biomaterials reduces adverse effects
Cu	Upregulates VEGF expression via ROS signaling (H_2_O_2_), supports EC migration and proliferation, and catalyzes pro-angiogenic reactions	↑ VEGF, FGF-1, angiogenin, HIF-1α, eNOS; enhanced VEGF in keratinocytes with ROS interaction	Excess causes oxidative stress; essential in small amounts but toxic at high concentrations
Ti	Surface topography influences osteoblast and EC response; activates integrin-dependent signaling pathways	↑ VEGF-A, FGF-2, ANG-1, VEGFR-1/2, eNOS, iNOS; enhanced NO production and EC activation	Low inherent toxicity: roughened surfaces increase bioactivity without raising toxicity

**Table 3:** Classification of the collected publications according to the angiogenic mechanisms of cobalt ions.

Mechanism	Description	Source
Stabilization of HIF-1α	Co^2+^, as an inducer of the hypoxic response, triggers a coordinated activation of a wide array of HIF-dependent genes, mostly VEGF	[1,4,5,6,7,8,9,10,11,12,13,14,20,27,82,83,84,85,86,87,88,89,90]
Induction of heme oxygenase-1 (HO-1) expression	Co^2+^ significantly upregulated VEGF and IL-8 via HO-1 activation, independently of the HIF-pathway	[88]
Influence on ECs causing high integrin-β1 expression	Integrin-β1 plays a crucial role in cell–cell and cell–matrix interactions, highly influencing angiogenesis	[9,27]

## Data Availability

Data sharing is not applicable to this article, as no datasets were generated or analyzed during the current study.

## Abbreviations

The following abbreviations are used in this manuscript:

Abbreviation	Full Term
ACGIH	The American Conference of Governmental Industrial Hygienists
Ade	Adenosine
aFGF/bFGF	Acidic/Basic Fibroblast Growth Factor
ANG-1	Angiopoietin-1
BAX	Bcl-2-Associated X Protein
BH4	Tetrahydrobiopterin 4
BMSCs	Bone Marrow Stromal Cells
CaP	Calcium Phosphate
Cbl	Cobalamin (Vitamin B_12_)
CD133^+^	Umbilical Cord Blood-Derived Cells
Co	Cobalt
Co^2+^	Cobalt Ion
CoCl_2_	Cobalt Chloride
Co-MMSNs	Cobalt-Doped Mesoporous Silica-Coated Magnetic Nanoparticles
CoPPIX	Cobalt Protoporphyrin IX
Cu	Copper
DMT1	Divalent Metal Transporter 1
DOAJ	Directory of Open-Access Journals
ECs	Endothelial Cells
ELISA	Enzyme-Linked Immunosorbent Assay
eNOS	Endothelial Nitric Oxide Synthase
EPO	Erythropoietin
ERK1/2	Extracellular Signal-Regulated Kinases 1 and 2
FAD	Flavin Adenine Dinucleotide
FGF	Fibroblast Growth Factor
FMN	Flavin Mononucleotide
GLUT1/3	Glucose Transporter 1/3
GSCbl	Glutathionylcobalamin
GSH	Glutathione
H_2_O_2_	Hydrogen Peroxide
hBMSCs	Human Bone-Marrow-Derived Stem Cells
Hcy	Homocysteine
HDAC6	Histone Deacetylase 6
HIF	Hypoxia-Inducible Factor
HIF-1α	Hypoxia-Inducible Factor 1-Alpha
HIF-1β	Hypoxia-Inducible Factor 1-Beta
HMEC-1	Human Microvascular Endothelial Cells
HO-1	Heme Oxygenase-1
HUVECs	Human Umbilical Vein Endothelial Cells
IL-8	Interleukin-8
iNOS	Inducible Nitric Oxide Synthase
LD	Linear Dichroism
MBG	Mesoporous Bioactive Glass
MCM	Methylmalonyl-CoA Mutase
MDPI	Multidisciplinary Digital Publishing Institute
MS	Methionine Synthase
NADPH	Nicotinamide Adenine Dinucleotide Phosphate
NO	Nitric Oxide
NOAEL	No-Observed-Adverse-Effect Level
NTP	National Toxicology Program
PDGF	Platelet-Derived Growth Factor
PLA	Poly (Lactic Acid)
qPCR/RT-qPCR	Quantitative Real-Time PCR
RfD	Reference Dose
ROS	Reactive Oxygen Species
TCN2	Transcobalamin II
TET	Ten-Eleven Translocation Methylcytosine Dioxygenase
TGF-β	Transforming Growth Factor Beta
Ti	Titanium
TLA	Three-Letter Acronym
TLV	Threshold Limit Value
VE-cadherin	Vascular Endothelial Cadherin
VEGF	Vascular Endothelial Growth Factor
VEGFR-1/2	Vascular Endothelial Growth Factor Receptor 1/2
vWF	von Willebrand Factor
